# Towards the computational design of protein post-translational regulation

**DOI:** 10.1016/j.bmc.2015.04.056

**Published:** 2015-06-15

**Authors:** Marta Strumillo, Pedro Beltrao

**Affiliations:** a European Molecular Biology Laboratory, European Bioinformatics Institute, Wellcome Trust Genome Campus, Cambridge CB10 1SD, UK; b iBiMED and Department of Health Sciences, University of Aveiro, 3810-193 Aveiro, Portugal

**Keywords:** Bioinformatics, PTMs, Allosteric

## Abstract

Protein post-translational modifications (PTMs) are a fast and versatility mechanism used by the cell to regulate the function of proteins in response to changing conditions. PTMs can alter the activity of proteins by allosteric regulation or by controlling protein interactions, localization and abundance. Recent advances in proteomics have revealed the extent of regulation by PTMs and the different mechanisms used in nature to exert control over protein function via PTMs. These developments can serve as the foundation for the rational design of protein regulation. Here we review the advances in methods to determine the function of PTMs, protein allosteric control and examples of rational design of PTM regulation. These advances create an opportunity to move synthetic biology forward by making use of a level of regulation that is of yet unexplored.

## Graphical abstract

**Figure:**
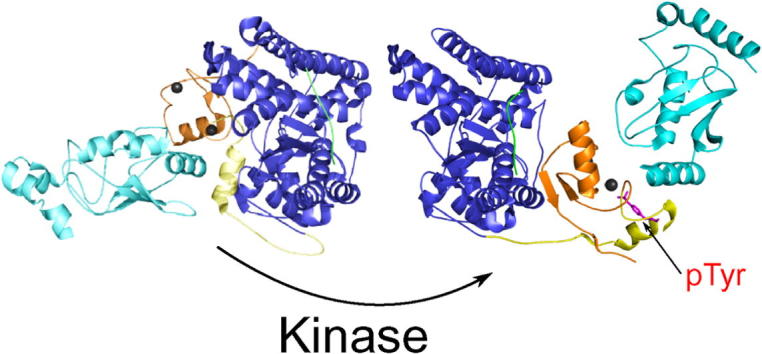

## 1 Introduction

The activity and function of proteins is finely tuned inside the cell in response to changes in internal and external conditions. Once a protein is expressed its function can be regulated by interactions with other proteins or with small-molecules. These interactions can cause the protein to be altered in different ways such as changing its localization or activity. Protein post-translational modifications (PTMs) are a common mechanism to regulate protein function. Examples of well-studied and highly abundant PTMs include phosphorylation, acetylation and ubiquitylation (reviewed in Refs. 1, 2) that alter the physicochemical properties of proteins. Each type of PTM is usually associated with a set of enzymes that can catalyse the addition or removal of the modification. For the three above mentioned PTMs these would correspond to protein kinases and phosphatases, lysine acetylases and deacetylases and ubiquitin ligases and de-ubiquitylases. PTMs can also occur without the need of protein enzymes. For example, it has been shown that acetyl-phosphate (AcP) can acetylate in vitro many proteins of the *Escherichia coli* proteome and that the levels of lysine acetylation in vivo are affected by the metabolism of AcP.^3^ Regardless of the process that causes the modification, PTMs can alter the function of proteins in many different ways. They are often found in interface regions^4^ and are known to tune the binding affinity of protein–protein interactions. The modification of localization signals can reversely regulate protein localizations^5^ and in addition PTMs are often able to regulate protein activities via allosteric regulation.^6^ For example, the modification of protein kinases or chaperones can allosterically control the activity of these enzymes.^7^, ^8^

In the past decade, advances in mass-spectrometry and enrichment strategies have increased the capacity to identify PTMs for different modification types.^2^ In a recent analysis of human proteins, Sharma and colleagues were able to identify over 50,000 phosphosites collected in one experiment.^9^ Compilation of different works have identified over 200,000 phosphosites, 35,000 acetylation sites and 50,000 ubiquitylation sites for human proteins alone (www.phosphosite.org). These experiments have revealed the large extent by which the proteome is modified and how much PTMs may be modulating protein function. The accumulation of PTM information for different species has also allowed for the study of their evolutionary properties.^10^, ^11^, ^12^, ^13^ Perhaps surprisingly, it was noted that many commonly studied PTM types tend to be poorly constrained leading some to suggest that a fraction PTM sites may serve no biological purpose.^12^ This would be analogous to the potential lack of function for many transcription-factor binding events in the genome.^14^ Given the large number of novel PTMs discovered and the lack of conservation it has been increasingly important to develop approaches to study PTM function^15^, ^16^, ^17^ (reviewed in Ref. 18). As we systematically characterize the naturally occurring modes of PTM regulation we should be able to extract rules to use for engineering via rational design.

Rational protein design has long history stemming from early work in protein computational modelling (reviewed in Ref. 19). Protein design approaches use similar computational methods as used in protein modelling but with the aim of finding the correct sequence that would fold into the target structure. There has been steady progress made in this field with successful designs of small proteins, initially based on naturally occurring folds^20^, ^21^ and later also applied to the design of novel folds.^22^ The same methods have been used also to engineer protein–protein interactions^23^, ^24^—reviewed in Ref. 25—as well as in enzyme design (reviewed in Ref. 26). We suggest that the large scale characterization of PTM function in natural systems is opening the door to the rational design of such regulatory events. Designed PTM regulatory systems could then be used in higher-order circuits in much the same way that transcriptional regulatory systems have been put together in synthetic biology to accomplish complex tasks. In this perspective we review the computational and experimental approaches used to determine the function of PTMs, the regions in proteins amenable for allosteric control as well as some seminal examples of rational design of PTM regulation.

## 2 Engineered regulation of interactions, localization and degradation

### 2.1 Design of domain–peptide interactions mediated by PTMs

**Figure 1:**
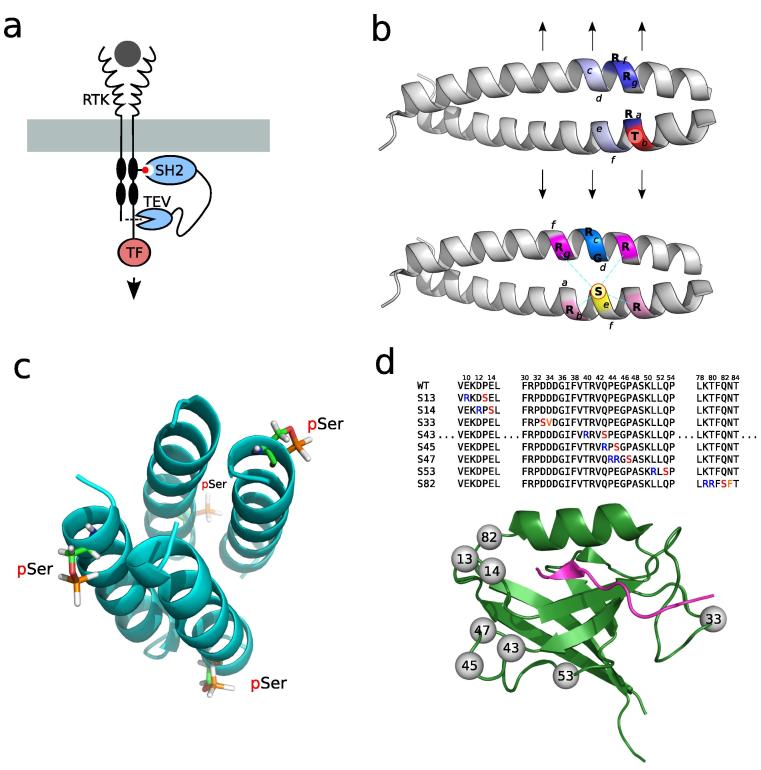
Design of post-translational regulation of protein–protein interactions. (a) The binding of phosphorylation ‘reader’ domains, like the SH2 domain, to their target peptides has been well characterized for some proteins. These rules can be used to engineer novel PTM regulated interactions like the recruitment of the TEV protease to an active RTK. Activation of the receptor causes the phosphorylation of specific tyrosine residues that recruit the binding of the SH2 domain. The recruited TEV can cleave off a transcription factor causing a downstream gene-expression response. (b) The leucine zipper pair illustrated here is a model for studying dimerization. Szilák and colleagues used this model system to study how the introduction of phosphorylation sites at different positions could regulate this protein interaction. The lower-case letters indicate the positions along the helix. In the example described in the upper diagram the phosphosite was introduced at a position ‘b’ (coloured red) that points way from the interface but causes a destabilization of the helix (at the positions coloured blue) and therefore inhibit the dimmer formation (arrows). The lower diagram illustrates the addition of a phosphosite at position ‘e’ (coloured yellow) pointing towards the interface where the phosphosite can interact with two opposing arginines (coloured magenta) and stabilize the interaction. (c) The Lac repressor oligomerization domain is another model system used to study protein–protein interactions. Signarvic and DeGrado found that phosphosites introduced in the monomer towards the N-terminus, illustrated here, caused an increase in the stability of the tetramer. (d) The structure of a Erbin PDZ (PDB: 1MFG) bound to a target peptide is shown. The positions highlighted were selected for the introduction of an engineered target site for the PKA kinase. The corresponding mutations needed to create a PKA target site are shown in the sequences above the structure.

Protein–protein interactions can very often be modulated by PTMs. The most commonly studied examples are from PTM recognition domains that are also known as ‘reader’ domains for their capacity to read the modification state of a protein. Each PTM type is often recognized by different protein domain families.^27^ For example, the SH2 domain family can recognize tyrosine phosphopeptides while the 14-3-3 domains bind to serine and threonine phosphosites. Similarly, Bromo domains recognize acetylation sites and Ubiquitin binding domains recognize ubiquitin. Like the enzyme regulators, each particular domain from a domain family has preferences for specific residues surrounding the target PTM site. These can be determined in large scale using different experimental approaches like peptide or protein arrays, phage display and mass spectrometry (reviewed in Ref. 28). Such studies have been applied extensively only for a small number of domain families such as the SH2,^29^ Polo-box,^30^ malignant brain tumor (MBT)^31^ and Bromo domains.^32^ Such studies define the sequence rules that, in part, determine the interaction between these proteins and modified peptides that exist in the context of full proteins.^33^ These rules can then be used to design PTM mediated interactions for desired outcomes. For example, Barnea and colleagues designed a receptor tyrosine kinase (RTK) reporter system using an SH2–phophotyrosine interaction^34^ (Fig. 1a). In this study the TEV protease was fused to an SH2 domain that can bind to auto-phosphorylated RTKs. They then fused a transcription factor to the cytosolic tail of the RTK with a linker that contained a TEV cleavage site. In this way, after RTK stimulation and auto-phosphorylation the protease gets recruited to the receptor by the phospho-dependent SH2 interaction. The protease cleavage releases the transcription factor that can elicit a signalling response (Fig. 1a). A similar strategy has been used to recruit a death effector domain to activated RTKs and in this way re-direct signalling pathways towards the induction of caspase activation and cell death.^35^ Engineered domain–peptide interactions have also been used to design kinase sensors^36^, ^37^, ^38^, ^39^ using a general strategy where two different fluorescent domains are joined by an intermediate section containing a peptide substrate for a kinase of interest a flexible linker and phospho-binding domain (e.g. SH2 or 14-3-3 domain). Upon phosphorylation the intra-molecular interaction decreases the distance between the fluorescence domains and increases the efficiency of fluorescence resonance energy transfer (FRET).

### 2.2 Tuning of protein–protein interfaces by PTMs

In addition to regulating interactions through recruitment of binding domains, PTMs can also tune the binding affinity of protein interactions by changing the properties of interfaces between globular domains.^4^, ^15^ Structural analysis has recently shown that protein–protein interfaces are enriched for modification sites.^4^, ^40^ In silico mutagenesis of these regulated interface positions suggests that many are likely to alter the binding affinity of the interaction.^4^ In addition, interface phosphosites and acetylation sites show an above average conservation suggesting that they are more likely than average sites to be evolutionarily constrained and therefore functionally important.^15^ These analyses suggest that PTMs often tune binding affinity in natural systems. This opens the possibility of using computational methods to redesign protein interfaces in order to add PTM regulation.

Computational design of interfaces has been used extensively in the past (reviewed in Ref. 25). Such efforts have been directed at improving binding affinity of existing interfaces as well as changing binding specificity. For example, Kapp and colleagues have developed a design strategy to create non-crossreacting (orthogonal) protein–protein interfaces.^23^ The authors developed this approach using, as a model interface, the interaction between the Cdc42 GTPase and Intersection (ITSN) its cognate GTPase exchange factor (GEF). Copies of these proteins were mutated in order to create a Cdc42–ITSN interface that would be functional but would not cross-react with the original pair. Perhaps the ultimate challenge in such design efforts is to be able to engineer a novel interface that does not exist in nature, something that was first achieved by Karanicolas and colleagues using a combination of computational design and directed evolution.^41^

The design of PTM regulation of protein interfaces has not yet been extensively explored. Seminal studies of engineered PTMs at interfaces come from the structural analysis of small model peptide systems. Szilák and colleagues, for example, used the leucine zipper coiled coil dimer of a bZIP DNA binding protein to study the effects of adding a phosphorylation site at different positions within its sequence (Fig. 1b). They showed that depending on the position the phosphosite can have both the capacity to destabilize^42^ or promote^43^ the association between the dimmer. The addition of phosphorylable positions was achieved by adding a PKA kinase recognition motif sequence in the desired positions. In one of the designs (Fig. 1b, lower diagram), the PKA motif was introduced such that the phosphosite would establish electrostatic interactions with two opposite arginines and in this way promote the dimmer formation. In the second design (Fig. 1b, upper diagram) the introduced phosphosite tends to destabilize the helix structure and in this way inhibit the formation of the dimmer. Similar work has been done to engineer a phosphorylation-dependent oligomerization domain based on the Lac repressor oligomerization domain.^44^ Signarvic and DeGrado engineered a 20-residue sequence such that 4 unstructured peptides monomers would create a four-helical bundle due to phosphorylation by the cyclic AMP-dependent protein kinase (PKA) (Fig. 1c). Beginning with the Lac repressor oligomerization domain, a phosphosite was introduced near the N-terminus leading to a 2–4.6 kcal/mol increase in the stability of the tetramer (Fig. 1c). More recently, computational tools were used to design a phosphorylatable PDZ domain resulting in changes in binding affinity towards a PDZ target peptide.^45^ Predictors of PKA substrate recognition were combined with the Rosetta software to scan the Erbin PDZ domains for positions that would be recognized by PKA without causing a large destabilization of the domain. Several designs were tested experimentally (Fig. 1d) with some resulting in changes in binding affinity and/or specificity.

### 2.3 Design of tunable protein localization and degradation

Protein localization and degradation are often also controlled by PTMs. Degradation in particular is often determined by ubiquitylation. Protein ubiquitylation in turn can be controlled by phosphorylation by what is commonly known as phospho-degron motifs.^46^, ^47^, ^48^ The phosphorylation of specific peptide sequences can result in the recruitment of ubiquitin ligases that have phospho-binding domains causing the ubiquitylation of nearby lysines. A few examples of phospho-dependent ubiquitylation events have been studied in detail to determine the rules required for the creation of a phospho-degron. These sequence motifs can in principle be grafted onto any protein of interest causing the degradation rate of target protein to depend on a kinase activity. A similar approach could be used to engineered tunable localization by grafting localization sequence motifs that are controlled by PTMs.^5^ Grafting of tunable localization signals has been achieved already for nuclear localization signals that are controlled by small molecules^49^, ^50^ or light.^51^, ^52^ Once developed, these PTM dependent degradation or localization signals could serve as highly modular tags and used to build higher order biological ‘circuits’ as it has been done extensively in synthetic biology.^53^

## 3 Engineering protein allosteric control

### 3.1 Natural examples of allosteric regulation

**Figure 2:**
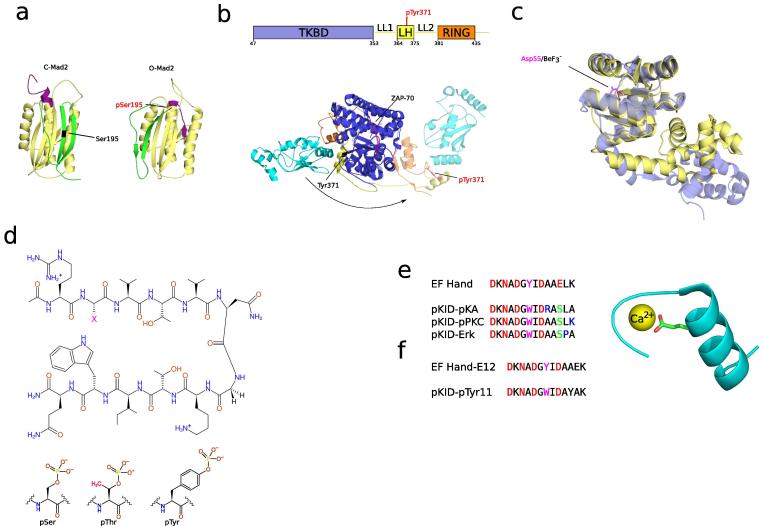
Allosteric regulation by post-translational modifications. (a) The structures of the open (O-Mad2, PDB: 1DUJ) and closed (C-Mad2, (PDB: 1S2H) conformations of the spindle checkpoint protein Mad2 are shown. The phosphorylation of serine 195 promotes the open conformation. The protein segments coloured in yellow show an overall similar conformation. Sections in green indicate the regions showing the highest change in conformation. The N-terminal region is highlighted in purple in both conformations. (b) The RING ubiquitin ligase c-Cbl contains 3 globular domains connected by linkers illustrated in the upper diagram. This protein can adopt two different conformations that are regulated by protein phosphorylation. In the unphosphorylated form the LH domain packs against the RING and TKBD domain (opaque structures, PDB: 1FBV). After phosphorylation LH and RING domain change their orientation relative to the TKBD domain drastically towards the binding interface of the target protein (PDB: 4A4C). This changes the orientation and distance between the E2 and the target protein. (c) The unphosphorylated (yellow, PDB: 4GVP) and beryllofluoride-actived (purple, PDB: 4IF4) conformations of the two-component response regulator VraR. Beryllofluoride (BeF3^−^) is mimicking phosphorylation. (d) Structure of a 12 residue peptide β-hairpin used to study the consequence of phosphorylation on allosteric regulation. The second position (X in the figure) of the hairpin was changed to different phosphosites and the impact on hairpin formation was evaluated. (e) and (f) The EF-hand helix-loop-helix structure was used to design a serine (e) and tyrosine (f) kinase sensor by introducing the phosphorylation sites highlighted green in the sequences. Evolutionary conserved glutamate at position 12 is highlighted in the sequences and structure.

Allosteric regulation is defined by the binding of another molecule or the PTM regulation of a region that is distinct from the active site of a protein. The modulation of protein function by allosteric mechanisms was the first mode of regulation by PTMs to be studied. Krebs and Fischer first discovered that the conversion between different states of glycogen phosphorylase was controlled by phosphorylation.^54^ Posterior structural work showed that the phosphorylation of a single residue (S14) results in a large change in the N-terminal region that changes the mode of binding of the dimer and subsequent enzyme activation.^55^ Since these seminal studies other proteins have been shown to be allosterically regulated by PTMs. For example, the spindle checkpoint protein Mad2 was shown to undergo a conformation transition that was dependent on the phosphorylation of S195^56^ (Fig. 2a).The c-terminal tail of Mad2 can be in two conformations, either exposing (open) or burying (closed) the c-terminal tail inside the protein. Phospho-S195 would be unfavourable in the closed conformation since it would place a negative charge inside the hydrophobic core of the protein. Kim and colleagues showed that the mutation S195D mimicked the phosphorylation of S195 and prevented the conformation transitions of Mad2, locking it in an open state.^56^ Single phosphorylation sites can cause extensive changes in protein conformation as seen in the regulation of the RING ubiquitin ligase c-Cbl^57^ (Fig. 2b). Like other Cbl proteins, c-Cbl contains a RING domain, an LHR domain and TKBD domain (Fig. 2b, upper diagram). The TKBD domain determines substrate specificity while the LHR and RING domains are important for the binding to E2 and catalysing the ubiquitylation of target substrates. c-Clb was shown to be regulated by phosphorylation in Y371 and detailed structural studies by Dou and colleagues have demonstrated that this is achieved via allosteric regulation. In the absence of the phosphosite c-Clb adopts a closed and auto-inhibit conformation where the RING domain interacts with the TKBD domain in way that inhibits the interaction between the E2 and the target protein (Fig. 2b). Phosphorylation of Y371 is not compatible with the closed conformation and abolishes the auto-inhibition of c-Clb.^57^ The open conformation results in a large rotation of the RING domain relative to the rest of the c-Clb protein bringing the E2 closer to the target protein (Fig. 2b). Understanding the allosteric regulation and conformation variability of proteins could be important for therapeutic purposes. For example, methicillin-resistant *Staphylococcus aureus* (MSRA) were found to be sensitive to deletion of the two component system VraSR.^58^ VraS and VraR form a two-component system composed of a regulator (VraS) that can act as a kinase or phosphatase for the transcription factor response partner VraR. Phosphorylation of VraR increases its transcriptional activity by an allosteric mechanism (Fig. 2c).^59^ Leonard and colleagues have shown that berylloflouride (BeF3^−^), acting as a phosphorylation mimic, opens the conformation of VraR (Fig. 2c) allowing for its dimerization and activation. Understanding the allosteric regulation of VraR by phosphorylation allows for the exploration of additional therapeutic strategies against MSRA.

### 3.2 Computational approaches to study allosteric regulation

The computational design of allosteric regulation requires a capacity to predict how a perturbation to one part of a protein, by a PTM or interaction, might result in a conformation change with impact on protein activity. One of the computational approaches used to study allosteric regulation is Molecular Dynamics (MD) that tries to simulate conformational changes over long timescales in protein structures. MD may often aid in the interpretation of existing structural information. For example, Espinoza-Fonseca and colleagues used MD to study the phospho-regulation of the N-terminal region of the regulatory light chain (RLC) of muscle myosin.^60^ This region is unstructured and to further study the dynamics of this regions MD simulations of the S19 phosphorylated and unphosphorylated suggested a phosphorylation induced disorder-to-order transition that was in-line with previous experimental data.^61^ The MD simulations provide the additional insight of producing atomic models of the potential different conformational states. Changes in conformation due to PTMs may also be important for drug interactions and drug design. This has been illustrated with the study of protonation driven changes in conformation of cathepsin B.^62^ Similar to PTMs, protonation status, due to change in pH, may alter the overall dynamics of the whole enzyme. Cathepsin B is inactivated in alkaline pH causing allosteric protection to the inhibitor heparin.^63^ Different pH conditions where simulated by Costa and colleagues and these simulations supported the hypothesis that the heparin binding to an allosteric site could stabilize the more stable conformation.^62^ These studies help to understand the mechanism activation of catB which might help in designing new catB inhibitors.

Other structure based computational approaches have been used to more directly attempt to identify allosteric ‘pathways’ or networks of residues that are important to communicate the information of a binding event or PTM. For example, Mitternach and Berezovky used Calpha elastic networks and normal mode analysis to identify potential protein motions and then used these to study the allosteric regulation by ligand binding or PTMs.^64^ An alternative approach was used by Cilia and colleagues whereby the conformation of side-chains across an NMR ensemble was probed using a rotomer library and the FoldX force field. These samples were then analysed to identify clusters of coupled side-chain that could predict residues involved in the long-range communication of the binding event for a PDZ domain.^65^

A sequence based computational approach that is commonly used to study allosteric regulation is protein sequence co-evolution. One of the more popular and successful of these algorithms for prediction of allosteric communication within proteins is the statistical coupling analysis (SCA).^66^ SCA measures the covariation between pairs of amino acids in large multiple sequence protein alignment of a given protein family. The sequence alignment contains the long-term evolutionary record of a protein family and SCA assumes that functionally relevant coupling between amino acids should drive co-evolution of those residues. Groups of contiguous co-evolving residues form networks or ‘sectors’ which are spatially organized within the protein and they can be linked to many surface sites distributed throughout the structure.^67^ These surface linked co-evolving sectors have been proposed as potential allosteric sites.^68^, ^69^ This approach has been used to study allosteric regulation in natural systems and although this has not yet been used to engineer PTM control it has been used to design a peptide binding domain^70^ and to guide allosteric drug design.^71^ Novinec and colleagues reported the discovery of a low-molecular-weight allosteric inhibitor of the collagenolytic cystein peptidase cathepsin K using SCA. In this study, the authors first predicted surface sites in the peptidase that are likely to be connected to the active site via networks of co-evolving sites. They then used high-throughput docking of compound libraries to these surface sites to find potential allosteric regulators.

### 3.3 Examples of engineered allosteric control by phosphorylation

The computational methods described above have yet not been applied to design PTM allosteric regulation. However, some authors have used short peptide systems to study the consequences of PTM regulation on protein conformation and in this way design PTM conformational switches. For example, Riemen and Waters analysed a 12 residue peptide designed to fold into a β-hairpin structure where the properties that determine the structure are well understood.^72^, ^73^ In one of the studies the second position of the hairpin was exchanged between a serine and different phosphosites (Fig. 2d). The introduction of any phosphosite at position 2 tended to destabilize the hairpin formation.^72^ A similar peptide system was also used to test the combination of phosphorylation and methylation on the stability of the β-hairpin structure.^73^ Another elegant example of controlled conformational switches was achieved by Balakrishnan and Zondlo making use of the EF-hand domain.^74^ The EF hand domain contains a calcium-binding motif that binds a metal atom, for example calcium in Calmodulin. However, due to similar electronics and ionic radii, EF-hands can also bind lanthanides, endowing these domains with luminescent properties that can be used as reporter systems. The authors engineered a protein kinase recognition sequence into an EF-hand and used luminescent as a readout for the folding of the domain. The EF-hand sequence is robust to mutations in different positions; however residue 12 that binds the metal ion in bidentate manner is invariantly Glutamic acid (Fig. 2e, EF Hand and structure). Binding of the ion to the EF-hand changes its conformation into a well-folded helix-loop-helix structure due to five side chain groups and one main chain carbonyl. The engineered EF-hand was built by modification of Glu in 12 position to phosphoserine creating motifs that are recognized by kinases (Fig. 2e, pKID-pKA, pKID-pPKC and pKID-ERK). The created domains act as a phosphorylation dependent switch that folds only when phosphorylated in position 12. A similar strategy was used to engineer an EF-hand that would fold after tyrosine phosphorylation.^75^ The authors reasoned that phosphotyrosine on the 11th position in EF-hand motif can replicate Glu in the 12th position given the large size of the tyrosine side-chain (Fig. 2f). These phosphorylation dependent EF-hands should be compatible with different kinase specificity preferences and might serve as modules to create allosteric regulation in other proteins by grafting them in appropriate locations.

## 4 Summary

The engineering of gene-expression regulatory circuits has already a long tradition (reviewed in Ref. 76). An increasing understanding of the design principles underlying natural systems and a well characterized list of components have allowed researchers to build circuits that achieve complex tasks (reviewed in Ref. 77). The design of biological circuits may one day allow for cell-based therapeutics that are not easily achieved with current conventional approaches.^78^ However, the engineering of post-translational control is currently lagging behind when compared to transcriptional regulation. Recent progress in proteomics is revealing the extent by which natural proteins are regulated by PTMs and in turn this is driving a renewed interest in approaches to determine the mechanisms by which different types of PTM exert their control. Here we have reviewed examples of the different modes of regulation by PTMs in natural systems, seminal works on the engineering of PTM regulation as well as computational tools that can be used for the computational design of PTM regulation. We think that the time is ripe for the development of parts and circuits based on PTM control. This will allow synthetic biology efforts to add regulation that can occur at faster time scales and through a much wider range of mechanisms.
